# Effectiveness of pharmaceutical care at discharge in the emergency department: study protocol of a randomized controlled trial

**DOI:** 10.1186/s13063-015-0579-3

**Published:** 2015-02-25

**Authors:** Regina Kuhmmer, Karine Margarites Lima, Rodrigo Antonini Ribeiro, Luciano Serpa Hammes, Gisele Alsina Nader Bastos, Maria Claudia Schardosim Cotta de Souza, Carisi Anne Polanczyk, Guilherme Alcides Flores Soares Rollin, Suhelen Caon, Cátia Moreira Guterres, Leni Everson Araújo Leite, Tássia Scholante Delabary, Maicon Falavigna

**Affiliations:** Institute for Education and Research, Hospital Moinhos de Vento, Rua Ramiro Barcellos 910, Bloco D, Porto Alegre, RS 90035-001 Brazil; Graduate Program in Cardiology and Cardiovascular Science, Universidade Federal do Rio Grande do Sul, Porto Alegre, Brazil; Graduate Program in Epidemiology, Universidade Federal do Rio Grande do Sul, Porto Alegre, Brazil; Department of Public Health, Universidade Federal de Ciências da Saúde de Porto Alegre, Porto Alegre, Brazil; Department of Clinical Epidemiology and Biostatistics, McMaster University, Hamilton, Canada

**Keywords:** Pharmaceutical care, Medication adherence, Blood pressure, Diabetes, Emergency

## Abstract

**Background:**

Patient education on pharmacological therapy may increase medication adherence and decrease hospitalizations. Our aim is to evaluate the effectiveness of pharmaceutical care at emergency department discharge in patients with hypertension and/or diabetes.

**Methods/design:**

This is a randomized controlled trial. Participants will be recruited from a public emergency department at Restinga district in Porto Alegre, southern Brazil. A total of 380 patients will be randomly assigned into 2 groups at the moment of emergency department discharge after receiving medical orientations: an intervention group, consisting of a structured individual counseling session by a pharmacist in addition to written orientations, or a control group, consisting only of written information about the disease. Outcomes will be assessed in an ambulatory visit 2 months after the randomization. The primary outcome is the proportion of patients with high medication adherence assessed using the Morisky-Green Test and the Brief Medication Questionnaire. The secondary outcomes are reduction of blood pressure, glycated hemoglobin, fasting plasma glucose, quality of life and number of visits to the emergency department.

**Discussion:**

Pharmaceutical care interventions have shown to be feasible and effective in increasing medication adherence in both hospital outpatient and community pharmacy settings. However, there have been no previous assessments of the effectiveness of pharmacy care interventions initiated in patients discharged from emergency departments. Our hypothesis is that pharmaceutical counseling is also effective in this population.

**Trial registration:**

ClinicalTrials.gov registration number: NCT01978925 (11 November 2013) and Brazilian Registry of Clinical Trials U1111-1149-8922 (5 November 2013).

**Electronic supplementary material:**

The online version of this article (doi:10.1186/s13063-015-0579-3) contains supplementary material, which is available to authorized users.

## Background

Cardiovascular disease (CVD) is the leading cause of mortality and burden of disease as measured in disability-adjusted life years (DALYs). Globally, high blood pressure and high blood glucose alone are responsible for 13% and 6% of deaths, respectively [1].

Treatment of chronic diseases commonly includes the long-term use of pharmacotherapy. Despite the efficacy of these treatments, inadequate medication adherence has been reported to approximately 50% of patients. Low adherence reduces the effectiveness of treatment, resulting in suboptimal illness control. As a consequence, an increased use of healthcare resources and reduction in patients’ quality of life has been observed [2-5]. About 20% of hospitalized patients experience adverse events after discharge. However, it is estimated that 60% of these events could be prevented with the proper use of medications [6-9].

Interventions focused on patient education related to pharmacological therapy may increase medication adherence and decrease morbidity [10]. Several interventions for improving adherence have been tested in clinical studies, including motivational and behavior strategies, simplification of dosing regimens, unit dose packaging, educational counseling, refill reminders and self-monitoring [11-16]. A systematic review of studies assessing interventions to improve medication adherence has shown an overall increase of 4 to 11%; however, no single strategy appeared to be superior [9].

Pharmaceutical care interventions for improving medication adherence seem to be feasible and effective, both in hospital and in community pharmacy settings [17,18]. Clinical pharmacists are able to identify and intervene to prevent potential problems with prescriptions written prior to discharge from the hospital, and studies have suggested that discharge counseling is able to improve medication adherence [19]. However, there is no evidence in the literature regarding the effectiveness of pharmaceutical care interventions initiated in patients discharged from emergency departaments (ED). In addition, few studies have assessed the impact of these interventions in developing countries, and the effect of these interventions in populations with lower income and educational level is uncertain.

The aim of this study is to compare pharmaceutical care at discharge from the ED with usual care in the adherence to medication prescriptions and in the control of hypertension and/or diabetes.

## Methods/design

### Study design

**Figure 1:**
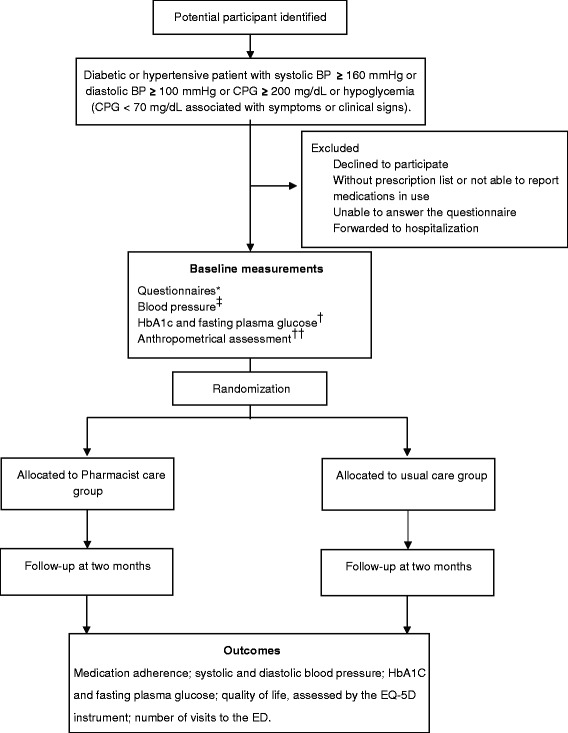
**Flow diagram of the study design.** *Questionnaires: sociodemographic variables; comorbidities; medication adherence; physical activity; alcohol consumption. ^‡^For patients with hypertension. ^†^For patients with diabetes. ^††^Anthropometrical assessment: weight, height, waist and hip circumferences. BP = blood pressure; CPG = capillary plasma glucose; HbA1c = glycated hemoglobin.

This is a randomized controlled, single-center study, with blinding of outcome assessors. A pilot study with ten patients was previously conducted in order to test study logistics and data collection instruments. Participants will be recruited from a public ED at Restinga district in Porto Alegre, southern Brazil. Porto Alegre is the 10th largest city in Brazil, with 1.5 million inhabitants. Restinga is a low-income district of Porto Alegre, with approximately 100,000 people and a Human Development Index (HDI) of 0.700 to 0.799, whereas Porto Alegre HDI ranges from 0.700 to 0.977 [20]. This ED is the single reference emergency service of the southern region of Porto Alegre, with 8,000 visits monthly. Eligible participants will be randomized to either the intervention or the control group, and will complete a follow-up interview after a period of 2 months (Figure 1).

### Inclusion and exclusion criteria

All patients with ED discharge from 8 am to 6 pm, on weekdays, will be screened for eligibility.

#### Inclusion criteria

- age ≥ 18 years;
- previous diagnosis and drug treatment for hypertension and/or diabetes;
- inadequate blood pressure (BP) control (systolic BP ≥ 160 mmHg or diastolic BP ≥ 100 mmHg), inadequate diabetes control (capillary plasma glucose ≥ 200 mg/dL) or moderate/severe hypoglycemia (capillary plasma glucose < 70 mg/dL associated with symptoms or clinical signs).

#### Exclusion criteria

- patients without a prescription list or not able to report medications in use;
- patients not residing in the city of Porto Alegre;
- patients unable to answer the questionnaire or to sign the informed consent form;
- patients forwarded to hospitalization.

### Study conduct

Eligible participants will be identified through active screening by a research assistant, reviewing medical notes of admitted patients. The research assistant will describe the project to each potential participant, will provide a straightforward language statement and will invite them to participate in the study. If the participant accepts, written informed consent will be sought before baseline interview and physical examination. All participants will be referred to the study pharmacist for randomization after baseline data collection.

### Baseline measures

Data collection at baseline includes measurement of BP (for patients with hypertension), glycated hemoglobin (HbA1c) and fasting plasma glucose (for patients with diabetes). In addition, medication in use, socio-demographic, comorbidity and anthropometrical variables (for example, gender, age, ethnicity, marital and smoking status, alcohol consumption, physical activity, circumferences and body mass index) will be recorded. The medical prescription at discharge will also be documented.

### Randomization and participants allocation

Following baseline data collection, eligible participants will be randomized to either the intervention or the control group in a 1:1 ratio, using a telephonic central for allocation concealment. The randomization sequence will be generated by using a computer program in blocks of four, six and eight. Randomization will be stratified according to the number of different medications in use (<5 or ≥ 5).

### Pharmaceutical care group

In the ED, immediately after discharge, participants randomized to the pharmaceutical care group will receive intervention coordinated by the study pharmacist. The clinical pharmacist will provide a structured 30-minute intervention for enhancing their medication adherence.

The recommendations include: discussion on hypertension and/or diabetes, risk of complications, prescribed drug therapy, correct use of medications and proper dosage, possible adverse effects, route of administration, schedule of administration and correct storage. The pharmacist will also emphasize the importance of lifestyle modifications. Printed educational material, with information on hypertension and/or diabetes medications, including suggested lifestyle interventions (for example, reduce salt and sugar intake, practice regular physical activity, smoking cessation, reducing alcohol consumption, monitor stress levels in day-to-day and reduce weight and keep it within the normal range) was prepared to assist in the intervention and will be handed to patients in the end of the session.

### Control group

In addition to counseling provided by a physician and by the nursing staff during their stay in the ED (usual care), patients randomized to the control group will receive the same printed material information on hypertension and/or diabetes medications and lifestyle interventions in order to keep patients masked.

### Follow-up visit

An ambulatory follow-up visit will occur 2 months after randomization for the assessment of the study’s outcomes. We will contact participants by phone prior to the follow-up visit in order to minimize losses; if necessary, home visits will be performed for those without conditions to attend the follow-up visit in the clinic. Data will be collected by a research assistant who is unaware of the randomization status.

### Primary endpoint

The primary outcome is the proportion of patients with high medication adherence at 2 months. Medication adherence will be assessed subjectively using two validated questionnaires in the Portuguese language: the Morisky-Green Test (MGT) and Brief Medication Questionnaire (BMQ) [21,22]. The MGT assesses both intentional and unintentional nonadherence and comprises four items. Responses for each item are scored one for ‘yes’ and zero for ‘no’ and are added together. A total score of zero represents good adherence and a score of one or more represents suboptimal adherence. The BMQ is composed of eleven questions, divided into three domains (regime, beliefs and memory).

### Secondary endpoints

The secondary outcomes are:

- systolic and diastolic BP, for patients with diagnosis of hypertension;
- HbA1c and fasting plasma glucose, for patients with diagnosis of diabetes;
- quality of life, assessed by the the EuroQol five-dimension (EQ-5D) instrument [23,24];
- number of visits to the ED.

### Sample size

The sample size was estimated assuming a percentage of 50% nonadherence to the medication treatment in the control group [25]. To detect a 15% absolute increase in the medication adherence in the intervention group, a sample size of 190 participants will be needed in each group, considering an alpha error of 5% (bilateral), statistical power of 80% and 10% of losses to follow-up.

One of the researchers of the study will be responsible for quality control of the collected information. The participants will be randomly selected to answer a simplified questionnaire containing key issues by telephone. This control will be performed in 10% of the sample.

### Data analysis

The questionnaires will be entered into the database through processing by optical reader, using the Remark Office OMR® Software (version 8.0 Gravic, Inc., Malvern, PA) . All analyses will follow the intention-to-treat (ITT) principle. For the primary outcome, we will initially consider in the analysis patients with complete follow-up. A secondary analysis will be performed with data of all patients initially randomized, considering, as the worst plausible scenario, that 29% of the losses to follow-up, in both groups, are adherent. The worst plausible scenario was based on a previous observational study, conducted in 2012 in the same region, showing that 34.5% (95% confidence intervals (CI), 29% to 40%) of patients with hypertension assisted in basic health units are adherent according to the same instrument [26]. We will not use any imputation method for continuous outcomes, such as BP and blood glucose. For these outcomes, we will include only those participants who attended the follow-up visit.

Data will be presented as relative risk (RR) and mean difference (MD), with 95% CI. Categorical variables will be compared using the chi-square test; continuous variables will be compared using the Student *t-*test or the Mann–Whitney *U-*test. The analysis will be conducted in SPSS for Windows (version 19.0, Redmond, WA, USA) and Stata (version 10, College Station, TX, USA); the statistician will be blinded for the randomization status.

### Ethics

This study has been approved by the Institutional Review Board of Hospital Moinhos de Vento of Porto Alegre (approval number 403.658). Written informed consent will be obtained from each participant at the time of enrolment. The trial has also been registered in the Clinical Trial Registry NCT01978925 and in the Brazilian Registry of Clinical Trials U1111-1149-8922, and according to the Brazilian Ethics in Human Research Regulations.

## Discussion

We present a study protocol for a randomized controlled trial to investigate the impact of pharmaceutical care in patients with chronic conditions at discharge from an ED. The study will be conducted in a setting with social vulnerability in a developing country. We already knew that at least half of the people in treatment for hypertension and/or diabetes in the same community do not adhere adequately to medications. As consequence, many would develop undesirable outcomes, such as clinical complications and increase in the use of health services resources that could be avoided with adequate adherence to the medical prescription. The moment of the discharge from the ED may constitute an important opportunity for medication reconciliation, reviewing and providing information related to the medications of continuous use [27,28]. In this context, we are proposing an intervention focused on pharmaceutical care at emergency discharge in order to enhance adequate medication use. Our hypothesis is that patients receiving pharmaceutical orientation in the intervention group will present better adherence to pharmacological therapy, resulting in better control of BP and glycemic profile in our study population.

Several studies have shown that pharmaceutical care in the treatment of patients with hypertension and diabetes has been associated with significant reductions in hospitalizations, visits to the ED and increase in medication adherence [3,9,17-19]. These studies were conducted mainly in hospital and ambulatory settings in developed countries. To our knowledge, this study is the first designed to evaluate the effectiveness of pharmaceutical care interventions in patients discharged from a public ED. With this randomized controlled trial we expect to provide evidence regarding the effectiveness of pharmaceutical care for chronic conditions at ED discharge. As consequence, these results may be useful for policy -making related to the development and implementation of pharmaceutical care programs in emergency services.

## Trial status

The trial is currently in the recruitment phase.

## Abbreviations

BMQ: Brief Medication Questionnaire
BP: blood pressure
CI: confidence interval
CVD: cardiovascular disease
DALY: disability-adjusted life year
ED: emergency department
EQ-5D: EuroQol five-dimension instrument
HbA1c: glycated hemoglobin
HDI: Human Development Index
ITT: intention-to-treat
MD: mean difference
MGT: Morisky-Green Test
RA: research assistant
RR: relative risk
